# Changes in CD73, CD39 and CD26 expression on T-lymphocytes of ANCA-associated vasculitis patients suggest impairment in adenosine generation and turn-over

**DOI:** 10.1038/s41598-017-12011-4

**Published:** 2017-09-15

**Authors:** Lovis Kling, Urs Benck, Annette Breedijk, Lisa Leikeim, Marianne Heitzmann, Stefan Porubsky, Bernhard K. Krämer, Benito A. Yard, Anna-Isabelle Kälsch

**Affiliations:** 1 Vth Department of Medicine (Nephrology/Endocrinology/Rheumatology), University Medical Centre Mannheim, University of Heidelberg, 68167 Mannheim, Germany; 2 Department of Pathology, University Medical Centre Mannheim, University of Heidelberg, 68167 Mannheim, Germany

## Abstract

Extracellular adenosine, generated via the concerted action of CD39 and CD73, contributes to T-cell differentiation and function. Adenosine concentrations are furthermore influenced by adenosine deaminase binding protein CD26. Because aberrant T-cell phenotypes had been reported in anti-neutrophil cytoplasmic auto-antibody (ANCA)-associated vasculitis (AAV) patients, an impaired expression of these molecules on T-cells of AAV patients was hypothesized in the present study. While in AAV patients (n = 29) CD26 was increased on CD4^+^ lymphocytes, CD39 and CD73 were generally reduced on patients’ T-cells. In CD4^+^ cells significant differences in CD73 expression were confined to memory CD45RA^-^ cells, while in CD4^-^ lymphocytes differences were significant in both naïve CD45RA^+^ and memory CD45RA^-^ cells. The percentage of CD4^-^CD73^+^ cells correlated with micro-RNA (miR)−31 expression, a putative regulator of factor inhibiting hypoxia-inducible factor 1 alpha (FIH-1), inversely with serum C-reactive protein (CRP) and positively with estimated glomerular filtration rate (eGFR). No correlation with disease activity, duration, and ANCA profile was found. It remains to be assessed if a decreased CD73 and CD39 expression underlies functional impairment of lymphocytes in AAV patients. Likewise, the relations between frequencies of CD4^-^CD73^+^ cells and serum CRP or eGFR require further functional elucidation.

## Introduction

There is compelling evidence that immune cell function and immune responses are heavily influenced by purinergic mediators. Following a variety of tissue insults, ATP is released into the extracellular space where it is rapidly converted to ADP, AMP and adenosine by the membrane associated ecto-nucleotidases CD39 and CD73^1,2^. Deamination of adenosine finally results in the generation of inosine and ammonia. Adenosine deaminase (ADA) deficiency is associated with severe combined immunodeficiency (SCID) underscoring its role in immune regulation^3^. Human ADA1 can bind dipeptidyl peptidase IV (CD26) on T-cells and may counteract regulatory T-cell-mediated T-cell suppression^4^. The local expression of CD73, CD39 and CD26 thus controls concentrations of ATP, ADP, AMP, adenosine and inosine in the extracellular space^5–8^.

While ATP promotes inflammation, T-cell activation and Th17 differentiation, adenosine controls vascular permeability and exerts immunosuppressive effects^9^ mainly through activation of A_2A_ receptors on immune cells^10,11^. Hypoxia strongly influences adenosine signalling to immune cells partly via stabilisation of hypoxia inducible factor-1α (HIF-1α), which in turn drives the induction of adenosine receptors and CD73^12^. Apart from hypoxia, CD73 is regulated by various cytokines, e.g. interferon(IFN)-β^13^, TGFβ, IFNγ, IL-6, and IL-12^14^, and by adenosine itself in a paracrine manner^15^. CD73 expression is influenced indirectly by factors that regulate factor inhibiting HIF-1α (FIH-1), e.g. micro-RNAs (miR)^16–19^. The importance of CD73 in cell mediated immunity has been appreciated amongst others in studies using CD73 deficient mice^20^ and models of auto-immune uveitis^21^. Other experimental studies have suggested CD73 as novel therapeutic target in cancer^22,23^, multiple sclerosis^24^, and chronic Toxoplasma gondii infection^25^.

Anti-neutrophil cytoplasmic auto-antibody (ANCA)-associated vasculitis (AAV), i.e. granulomatosis with polyangiitis (GPA), microscopic polyangiitis (MPA), eosinophilic granulomatosis with polyangiitis (EGPA) and renal-limited AAV^26^ share common histopathological findings of systemic small vessel vasculitis with fibrinoid necrosis of the vessel wall or necrotizing, crescentic glomerulonephritis^27,28^. While ANCA are believed to be directly involved in the pathogenesis of AAV, their presence suggests impaired regulation of immune tolerance. Because isotype switching from IgG_1_ to IgG_4_, the predominant IgG ANCA subclasses^29^, depends on T-cell cytokines, impaired regulation of immune tolerance in AAV patients may not be restricted to the B-cell compartment alone but likely involves T-cells as well. Indeed, aberrant T-cell phenotypes have been identified in AAV patients including expanded effector memory T-cells^30^, defective regulatory T-cells^31^ and expanded Th17 cells^32^. In keeping with these findings and the role of adenosine in immunity, we hypothesized that T-lymphocytes from AAV patients may disclose an altered expression of cell surface molecules involved in adenosine generation and turn-over. In addition, we assessed if this was associated with differential miR expression and to what extent an altered CD39 and CD73 expression correlated with clinical entities.

## Results

### CD73, CD39 and CD26 expression on T-cells of AAV patients

**Figure 1:**
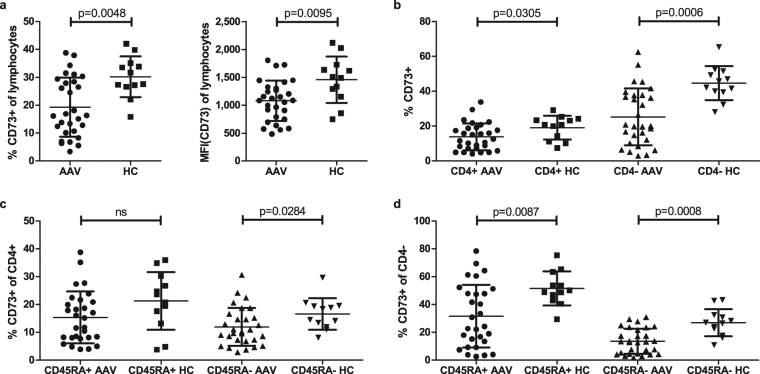
Reduction of CD73 frequency in lymphocytes from AAV patients. (**a**) CD73 frequency and protein level (median fluorescence intensity, MFI, given in relative light units, RLU) were significantly reduced in the entire lymphocytic population from ANCA patients. (**b**) CD73^+^ cells were reduced both in the CD4^+^ and CD4^-^ fraction in the patient cohort. (**c**) CD4^+^CD73^+^ populations were solely decreased in the memory (CD45RA^-^) subset from ANCA patients, while CD4^-^ CD73^+^ cells were significantly diminished in the memory and the naïve (CD45RA^+^) subpopulation as well (**d**). AAV: patient group, HC: control group. Non-parametric two-tailed Mann-Whitney-U-test was used to test for significance; ns: not significant.

**Figure 2:**
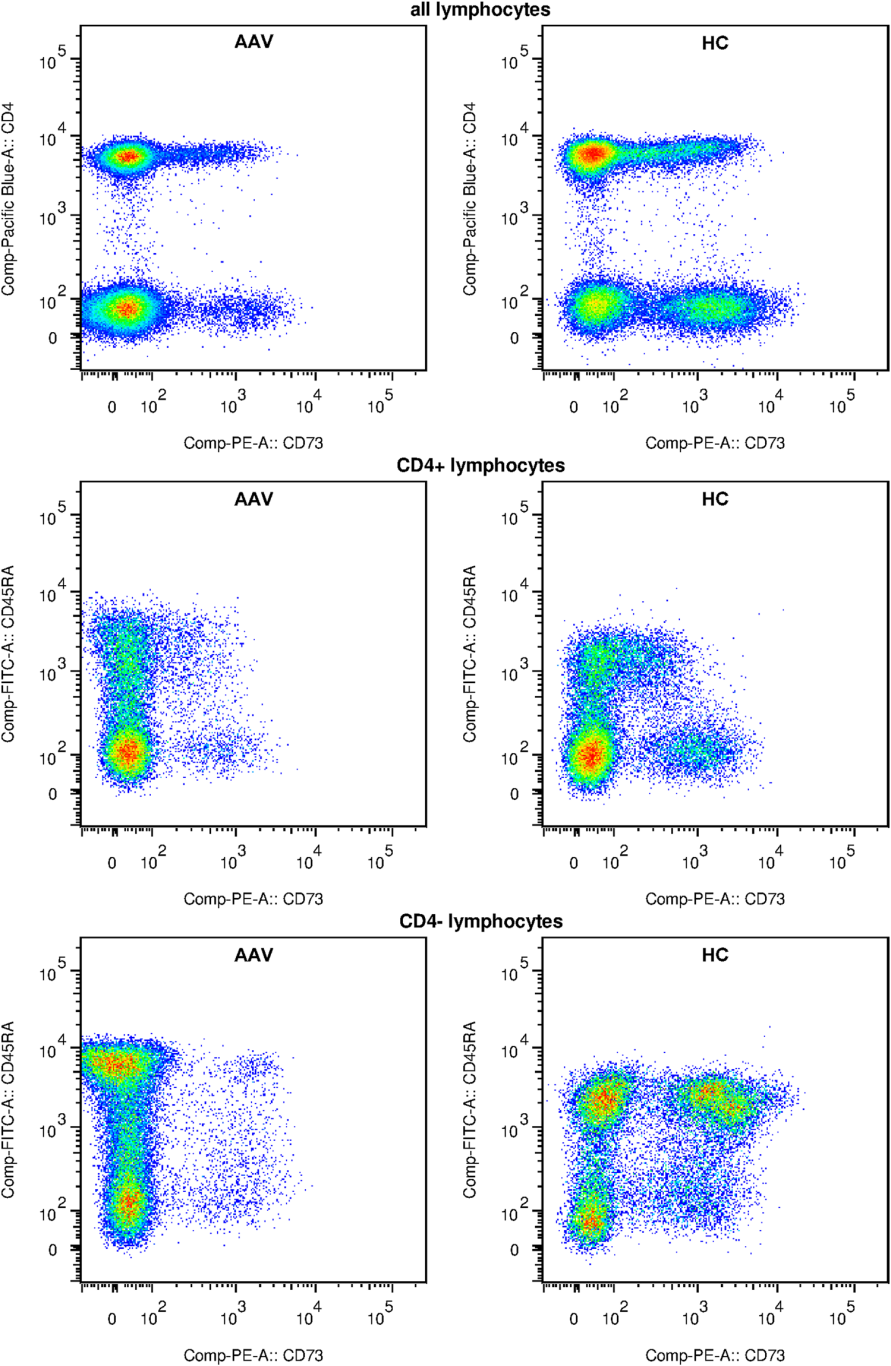
Exemplary CD73^+^ lymphocyte populations of one AAV patient (AAV, left column) compared to a healthy control (HC, right column). First row: all lymphocytes were gated, CD4 intensity is plotted against CD73. Only CD4^+^ lymphocytes (second row), and only CD4^-^ lymphocytes (third row) were gated, CD45RA intensity is plotted against CD73. Colours indicate cell density in descending order: red, yellow, green, blue.

CD73, CD39 and CD26 expression were assessed by FACS on peripheral blood lymphocytes of AAV patients (n = 29, 2 samples were not eligible for FACS analysis) and healthy controls (HC, n = 12). Demographic and clinical characteristics are depicted in Table 1 and in methods. Both the frequency and median fluorescence intensity (MFI) of CD73^+^ lymphocytes were significantly reduced in AAV patients as compared to HC (Fig. 1a). This was found in both CD4^-^ and CD4^+^ lymphocytes, albeit these differences were more pronounced in the former population (Fig. 1b). Differences in the frequency of CD73^+^ cells between patients and HC were significant within the CD4^+^ population only in the memory CD45RA^-^ lymphocytes (Fig. 1c), while in the CD4^-^ lymphocytes this was observed for both naïve CD45RA^+^ and memory CD45RA^-^ lymphocytes (Fig. 1d). Figure 2 shows illustrative dot plots from an exemplary AAV patient and HC for CD73 expression in all lymphocyte subsets. Expression differences for CD39 between patients and HC were confined to the CD4^-^ lymphocytes. CD4^-^CD45RA^+^ lymphocytes of patients showed a reduced frequency of CD39, yet CD39 MFI was increased on these cells. In contrast, CD39 MFI in the CD4^-^CD45RA^-^ lymphocytes of patients was reduced with no significant change in the percentage of CD39^+^ cells between patients and HC (Table 2). Differences in CD26 expression were only observed in CD4^+^ lymphocytes, in both the naïve and memory subsets (Table 2). When MFI for CD73, CD39 and CD26 was ranked from high to low, it was found that in patients MFI of CD26 was higher than that of CD73 (CD26 > CD73 > CD39; 1498 ± 506.8, 1082 ± 361.5 and 615.4 ± 172.1, respectively), while in HC CD73 was expressed stronger compared to CD26 (CD73 > CD26 > CD39; 1459 ± 415.1, 1173 ± 206.5 and 679.6 ± 190.8, respectively).

**Table 1:** Patient cohort characteristics. GPA: granulomatosis with polyangiitis, MPA: microscopic polyangiitis, IIF: indirect immunofluorescence of anti-neutrophil cytoplasmic antibodies, RTX: rituximab, CYC: cyclophosphamide, BVAS: Birmingham vasculitis activity score, GC: glucocorticoids, AZA: azathioprine, MTX: methotrexate, MMF: mycophenolate mofetil, COTRIM: trimethoprim/sulfomethoxazol, CRP: C-reactive protein in peripheral blood, eGFR: estimated glomerular filtration rate (MDRD formula), s.d.: standard deviation. Disease duration was defined as the period from initial diagnosis until study inclusion.

Diagnosis	Age	Gender	IIF	RTX/CYC in history	BVAS	Immunosuppression/Medication at point of study enrolment	Disease duration [months]	Time in remission [months]	Relapse rate [relapse/month]	CRP [mg/l]	Creatinine [mg/dl]/eGFR [ml/min]	Samples used in experiments
GPA	82	F	cANCA	RTX,CYC	0	GC	182.8	28.6	0.027	37.9	1.4/38	miR
MPA	75	F	pANCA	CYC	0	GC, AZA	170.6	64.1	0.006	<2.9	1.65/32	FACS, miR
GPA	75	M	cANCA	RTX, CYC	0	GC, COTRIM	243.9	9.3	0.004	17.6	4.27/14	FACS, miR
GPA	72	M	cANCA	RTX, CYC	29	GC	0.6	0	0	118	5.21/12	FACS, miR
GPA	21	M	cANCA	RTX, CYC	0	GC, COTRIM	51.6	2.9	0.019	<2.9	0.93/>60	FACS, miR
GPA	74	F	pANCA	RTX, CYC	0	GC	83	19.1	0.012	9	1.21/46	FACS, miR
MPA	78	M	pANCA	CYC	0	GC, AZA, COTRIM	40.4	40.4	0	7.5	0.89/>60	miR
GPA	24	F	cANCA	RTX	0	GC, COTRIM	11.4	11.4	0	<2.9	1.08/>60	FACS, miR
MPA	59	M	pANCA	—	0	GC, MTX	28.7	28.7	0	5	1.24/>60	FACS, miR
GPA	36	F	negative	—	0	GC, MTX	18	7.8	0.056	7.3	0.76/>60	FACS, miR
GPA	33	F	cANCA	RTX, CYC	0	GC	45.3	3.8	0.022	<2.9	1.29/50	FACS, miR
GPA	18	M	cANCA	RTX, CYC	0	GC, COTRIM	5	5	0	<2.9	0.87/>60	FACS, miR
GPA	75	F	cANCA	RTX, CYC	0	GC	205.9	17.2	0.024	24.5	0.76/>60	FACS, miR
GPA	55	F	cANCA	—	0	GC	165.6	92.5	0.012	41.3	2.82/19	FACS, miR
GPA	28	M	cANCA	RTX, CYC	0	GC, AZA	114.8	28.6	0.078	<2.9	0.91/>60	FACS, miR
MPA	69	M	cANCA	—	0	MMF	206.1	156.4	0.005	31.8	4.36/14	FACS, miR
GPA	75	M	pANCA	CYC	0	GC, AZA, COTRIM	45.2	45.2	0	9.4	1.51/48	FACS
GPA	65	F	pANCA	CYC	6	GC	88.9	0	0.011	14.2	0.75/>60	FACS, miR
GPA	82	M	cANCA	CYC	0	GC, MTX	187.8	187.8	0	<2.9	1.37/53	FACS, miR
GPA	80	M	cANCA	—	0	GC, AZA	70	70	0	<2.9	1.81/39	FACS, miR
MPA	67	F	pANCA	RTX	0	GC, COTRIM	18.4	18.4	0	18.7	2.45/21	FACS, miR
MPA	82	F	cANCA	RTX, CYC	0	GC, AZA	41.9	41.9	0	<2.9	1.28/42	FACS, miR
GPA	57	F	cANCA	—	2	GC, MTX	59.4	0	0	<2.9	0.96/>60	FACS, miR
MPA	86	F	cANCA	RTX, CYC	0	—	32	32	0	<2.9	1.38/39	FACS, miR
MPA	77	M	pANCA	—	18	GC	0.6	0	0	93.1	4.26/14	FACS, miR
GPA	62	M	cANCA	CYC	0	GC, AZA	61.6	61.6	0	<2.9	2.34/30	FACS, miR
GPA	52	M	cANCA	—	0	GC, COTRIM	14	7.9	0.071	15.2	1.65/47	FACS, miR
MPA	75	M	cANCA	CYC	0	GC, MMF	202.7	19.1	0.005	10.1	2.13/32	FACS, miR
GPA	61	M	cANCA	RTX, CYC	18	GC, AZA, COTRIM	132.9	0	0.015	<2.9	3.64/18	FACS, miR
GPA	53	M	cANCA	RTX	9	GC	0.3	0	0	3.6	0.87/>60	FACS, miR
MPA	62	M	pANCA	RTX, CYC	0	—	159.7	127.3	0.006	<2.9	1.91/38	FACS, miR
GPA: n = 21 MPA: n = 10	Mean ± s.d.: 69 ± 19.8 years	M: n = 17 F: n = 14	cANCA: n = 21 pANCA: n = 9 seronegative: n = 1	RTX: n = 16 CYC: n = 20			Mean ± s.d.: 86.8 ± 76.1 months	Mean ± s.d.: 36.4 ± 47.25 months	Mean ± s.d.: 0 ± 0.021 relapse/month	Mean ± s.d.: 27.3 ± 31.8 mg/l	Mean ± s.d.: Creatinine 1.87 ± 1.24 mg/dl eGFR 32.3 ± 13.7 ml/min	

Time in remission was calculated from the latest relapse, while recurrence of active disease requiring escalation of immunosuppressive treatment as compared to maintenance therapy defined relapse. Relapse rate was calculated from the number of relapses since the first manifestation of AAV.

**Table 2:** Altered expression of CD39 and CD26 in lymphocytic subpopulations from AAV patients.

	CD39						CD26					
	Patients		Controls		p-value		Patients		Controls		p-value	
	MFI [RLU]	%	MFI [RLU]	%	MFI	%	MFI [RLU]	%	MFI [RLU]	%	MFI	%
all lymphocytes	615.4 ± 172.1	9.41 ± 7.21	679.6 ± 190.8	14.15 ± 4.82	ns	0.0117	1498 ± 506.8	50.6 ± 16.78	1173 ± 206.5	59.76 ± 8.39	0.0264	ns
CD4+	531.6 ± 155.3	8.27 ± 5.25	532.7 ± 242.6	7.62 ± 6.04	ns	ns	1692 ± 559.4	79.73 ± 14.54	1200 ± 212	86.31 ± 3.72	0.0008	ns
CD4−	638 ± 250	10.59 ± 10.42	739.7 ± 180.6	22.01 ± 7.43	ns	0.0009	1154 ± 379.2	32.34 ± 16.52	1067 ± 250.4	31.67 ± 8.19	ns	ns
CD4+ CD45RA+	419.8 ± 182.4	2.53 ± 3.05	365.6 ± 136.3	2.38 ± 3.82	ns	ns	1558 ± 530.2	80.04 ± 20.71	1093 ± 230.6	90.83 ± 11.62	0.0025	0.0364
CD4+ CD45RA−	504.5 ± 144.9	13.07 ± 6.76	511.8 ± 223	11.79 ± 7.15	ns	ns	1714 ± 554.7	76.48 ± 13	1261 ± 205.9	81.31 ± 4.13	0.0018	ns
CD4− CD45RA+	726.3 ± 293.5	14.25 ± 15.4	373.9 ± 200.3	29.33 ± 8.37	0.0004	0.0019	1400 ± 784.8	23.14 ± 21.07	1465 ± 412.2	20.59 ± 12.64	ns	ns
CD4− CD45RA−	287.9 ± 72.2	4.53 ± 2.43	746.9 ± 173.7	4.14 ± 3.24	<0.0001	ns	1025 ± 503.5	80.77 ± 22.45	796.8 ± 149.7	93.47 ± 10.94	ns	ns

Sizes of different populations expressing CD39 and CD26, respectively, from AAV patients were compared to those from healthy controls. Population size is given in mean percentage ± standard deviation. Expression level of both proteins CD39 and CD26 is indicated by median fluorescence intensity (MFI) ± standard deviation. MFI was measured in relative light units [RLU]. Non-parametric two-tailed Mann-Whitney-U-test was used to test for significance; ns: not significant.

### CD4^-^CD73^+^ cells correlate with inflammation and renal function of AAV patients

**Figure 3:**
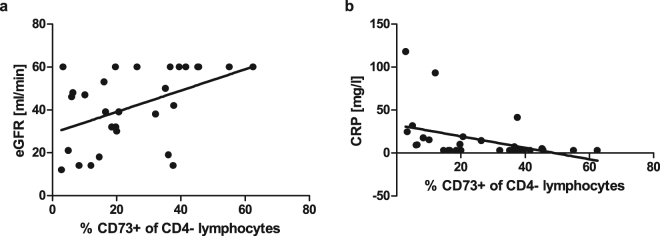
Correlation analysis of CD4^-^CD73^+^ lymphocytes with renal function and inflammation. **(a)** Frequency of CD4^-^CD73^+^ cells positively correlated with renal function assessed by eGFR; Spearman’s r = 0.492; P = 0.0067; R^2^ = 0.2137. **(b)** Frequency of CD4^-^CD73^+^ cells negatively correlated with CRP concentration in peripheral blood; Spearman’s r = −0.5081; P = 0.0049; R^2^ = 0.1657. Black lines indicate regression line.

We next explored if changes in T-cell subsets correlate with clinical entities, e.g. relapse rate, disease duration (time since initial diagnosis) and activity, ANCA profile, serum CRP and renal function assessed by estimated glomerular filtration rate (eGFR). Because for CD73, CD39 and CD26 expression on T-cells the difference between patients and HC were the strongest for the CD4^-^CD73^+^ T-cell subset, we chose the frequency of CD4^-^CD73^+^ T-cells as independent variable in univariate analysis. While no significant variation was evident between patients with active disease and in remission or different ANCA profiles as well as disease duration and relapse rate, the frequency of CD4^-^CD73^+^ T-cells disclosed an inverse correlation with serum CRP (r = −0.508; *P* = 0.0049, Fig. 3a) and was positively correlated with eGFR (r = 0.492; *P* = 0.0067, Fig. 3b).

### miR analysis by TaqMan Low Density Arrays

Since expression of miRs may influence the expression of CD73 directly or indirectly through HIF-1α or its repressor factor inhibiting HIF-1α (FIH1)^33,34^ we analysed miR expression in peripheral blood mononuclear cells (PBMC) of 20 patients (GPA: n = 16, MPA: n = 4) and 12 controls by means of TaqMan Low Density Array. We observed 29 miRs to be expressed significantly different when comparing AAV patients and HC (Table 3), while the vast majority of these miRs were up-regulated (fold change ranging from 1.37–2.69 in patients), only one miR, i.e. miR-31, was down-regulated (fold change 0.31 in patients) and found to be the most significant amongst the 29 differently expressed miRs.

**Table 3:** miR screening by TaqMan Low Density Arrays.

Up-regulation	Fold Change	Range	p-value
miR-193a-5p	1.37	0.91–2.07	0.047
miR-652	1.38	0.85–2.23	0.046
miR-93	1.39	0.96–2.01	0.041
miR-425	1.46	1.04–2.05	0.047
miR-671–3p	1.50	0.93–2.43	0.048
miR-484	1.53	1.18–1.99	0.019
miR-491–5p	1.60	1.16–2.22	0.031
miR-487a	1.60	0.99–2.59	0.05
miR-501–3p	1.62	1.02–2.58	0.048
miR-223	1.63	1.23–2.16	0.035
miR-133b	1.64	0.99–2.73	0.047
miR-574–3p	1.64	1.25–2.16	0.028
miR-579	1.66	1.01–2.73	0.037
miR-362–3p	1.67	1.08–2.58	0.05
miR-885–5p	1.69	1.06–2.70	0.029
miR-519d	1.70	1.04–2.77	0.041
miR-424	1.81	1.16–2.85	0.024
miR-886–5p	1.87	1.09–3.20	0.034
miR-191	1.87	1.30–2.69	0.021
miR-1	1.91	1.23–2.96	0.02
miR-200a	1.93	1.23–3.02	0.016
miR-302b	1.93	1.13–3.32	0.029
miR-107	1.96	1.25–3.05	0.03
miR-450b-3p	1.96	1.26–3.05	0.022
miR-582–5p	2.02	1.29–3.16	0.011
miR-145	2.09	1.11–3.93	0.045
miR-34a	2.50	1.63–3.83	0.021
miR-486–5p	2.70	1.3–5.59	0.039
Down-regulation			
miR-31	0.32	0.19–0.54	0.002

miR significantly altered in PBMC isolated from AAV patients (n = 20) as compared to healthy controls (n = 12). Mean fold change and its range in the patient group is listed.

### miR-31 correlates with lymphocytic CD73 frequency

**Figure 4:**
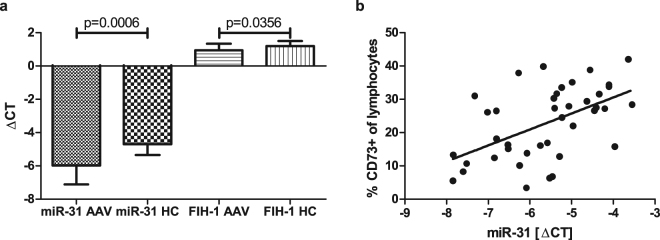
Significant down-regulation of miR-31 and its target FIH-1 mRNA in AAV patients. (**a**) miR-31 was repeatedly down-regulated in the patient group (AAV: n = 30, healthy controls: n = 12), so was its direct target FIH-1 mRNA as indicated by comparison of delta cycle threshold (ΔCT) values from qPCR between AAV patients and healthy controls. Non-parametric two-tailed Mann-Whitney-U-test was used to test for significance. **(b)** Quantity of miR-31 positively correlated with frequency of CD73^+^ lymphocytes in the entire study cohort (n = 40), Spearman’s r = 0.5433; P = 0.0003; R^2^ = 0.2838. Black lines indicate regression line.

As miR-31 was previously reported to be a direct target for FIH-1 mRNA^33^ and because HIF-1α is a transcription factor for CD73^12^, we performed qPCR for miR-31, HIF-1α and FIH-1 mRNA in our complete study cohort (AAV: n = 30, HC: n = 12). Indeed, we confirmed that miR-31 was significantly lower expressed in AAV patients (mean fold change ± s.d.: 0.55 ± 0.48), but unexpectedly, also its putative target FIH-1 mRNA was significantly down-regulated in the patient group (mean fold change ± s.d.: 0.87 ± 0.24) (Fig. 4a), whereas HIF-1α mRNA did not show significant discrepancy in the patient group (mean fold change ± s.d.: 0.81 ± 0.77, not significant). Nonetheless, the expression of miR-31 significantly correlated with the frequency of CD73^+^ T-cells when all study participants were analysed (Fig. 4b).

## Discussion

Our study demonstrates that the expression of CD73 and CD39, that are both implicated in the generation of extracellular adenosine, are down-regulated on T-cells of AAV patients, while the expression of CD26 on these cells is up-regulated. Since CD26 strongly associates with ADA, our findings may suggest an imbalance in T-cell mediated adenosine generation and turn-over in AAV patients. This may not be specific for AAV patients as serum ADA activity has also been reported to be increased in SLE patients^35^, while the expression of the adenosine-generating enzymes (CD39, CD73) seems to be decreased^36,37^. Most of the published studies on CD39 and CD73 expression on lymphocytes in relation to auto-immune diseases have focused on regulatory T-cells. In the current study, we demonstrate that CD39 and CD73 are expressed on all major T-cell subsets to a different extent and that differences between patients and HC in expression levels of these molecules unlikely are restricted to regulatory T-cells.

We are aware that CD73 expression on lymphocytes decreases with age^38^. Although this was also found in our study cohort, down-regulation of CD73 in AAV patients was still noticed when comparing individuals above 40 years of age in both groups.

We did not find correlations between CD73 expression and disease activity, relapse rate or time since initial diagnosis, yet, the relative number of CD4^-^CD73^+^ T-cells showed a correlation with serum CRP and renal function. An inverse correlation between CD73 and CRP has also been described in HIV patients. In these patients, depletion of CD4^+^CD73^+^ T-cells coincides with increased activation markers on CD4^+^ and CD8^+^ cells^39^ suggesting a role in persistent immune activation and inflammation. Nonetheless, we cannot exclude that CD39 and CD73 expression are influenced by immunosuppressive medication as has been demonstrated for methotrexate in the treatment of rheumatoid arthritis patients^40,41^ or azathioprine in inflammatory bowel disease patients^42,43^. Also rituximab and cyclophosphamide have been shown to modify peripheral T-cell subset frequencies^44,45^, but their effect on the expression of ecto-nucleotidases still remains elusive. Only four patients in complete remission, three presenting with normal and one with slightly reduced renal function, were treated with methotrexate and only two patients received mycophenolate mofetil. Even though AAV patients are relatively lymphopenic^46^, show persistent immune activation^47^ and may have signs of subclinical inflammation prior to relapses^48^, the cause of a diminished CD73^+^ T-cell population and its consequences in relation to inflammation and persistent immune activation remains to be assessed.

A relation between CD73 and reno-protection has been described in ischemia reperfusion injury (IRI) models in which adoptively transferred CD73^+^, but not CD73-deficient regulatory T-cells protect wild-type mice from kidney IRI^49^. Also, the glomerular filtration rate depends on CD73 derived adenosine as a main component of the tubulo-glomerular feedback^50^. In addition, CD73 deficiency appears to be associated with a vascular pro-inflammatory phenotype^51^, comprising the renal glomerular endothelium and interstitium^20^. Whether a decreased expression of CD73 in AAV patients is only confined to T-cells or also occurs in renal tissue is to our knowledge not known and subject of our currently ongoing research.

Inasmuch as miR expression, as part of epigenetic reprogramming, participates in T-cell activation and differentiation, dysregulation hereof may yield aberrant T-cell phenotypes contributing to auto-immunity^52^. In AAV patients, miR expression has been studied in the circulation^53,54^ and in peripheral blood mononuclear cells^55^. Amongst the significantly changed miR in our study, 3 miR are indirectly associated with CD73 expression, i.e. miR-31, miR-424 and miR-34a. While the latter 2 miR increase CD73 expression through HIF-1α up-regulation^56^ or inhibition of HIF-1α proteasomal degradation^57^, miR-31 might influence CD73 expression via targeting FIH-1 mRNA^33^. Since FIH-1 impedes the transcriptional activity of HIF-1α and thus transcription of CD73^34^, down-regulation of miR-31 may result in down-regulation of CD73. Indeed, the strong correlation with CD73 expression found in our study suggests a regulatory role for miR-31, yet this assumption awaits further experimental confirmation. It is noteworthy to mention that in addition to the findings of Li *et al*. on CD39 and CD73 expression on lymphocytes of SLE patients^36,37^, also in these patients, down-regulation of miR-31 has been reported.

Lymphocyte infiltrations in vascular lesions of AAV patients have been described in numerous studies^58,59^. Aberrant T-cell phenotypes, e.g. memory CD8^+^CD28^-^ cells which are believed to produce IFNγ and TNFα^60^, can be found in these infiltrates. It is therefore tempting to speculate that a relative shortage of adenosine, potentially caused by changes in CD26, CD39 and CD73 expression, may drive the pro-inflammatory milieu in lesions of AAV patients. The expression of CD26 in nasal biopsies of patients with localized GPA has been reported to be high^61^, yet the expression of CD39 and CD73 has not been studied in lesions of AAV patients.

In conclusion, the findings of our study suggest that T-cells from AAV patients may disclose an impaired ability to generate adenosine as a consequence of reduced expression of adenosine generating ecto-nucleotidases (CD39 and CD73) while at the same time an increased expression of the adenosine deaminase binding protein CD26 may favour adenosine turn-over. Our study, however, does not provide experimental evidence that adenosine concentrations are low in lesions of AAV patients and that this in turn underlies the pro-inflammatory milieu of such lesions. Nonetheless, the correlations found with CRP and renal function indicate a potential role for adenosine, but warrants further functional studies to underpin the clinical relevance of our findings.

## Methods

### Recruitment of study participants

Biopsy proven AAV patients (n = 31) treated in our department and healthy controls (n = 12, male: n = 6, female: n = 6, mean age ± standard deviation: 55.5 ± 16.1 years) were investigated in this study. For detailed patient cohort characteristics see Table 1. Diagnosis of AAV followed the Chapel Hill Nomenclature and diagnostic criteria of the American College of Rheumatology. Disease activity was assessed using the Birmingham Vasculitis Activity Score (BVAS) for EUVAS studies modified from Luqmani *et al*. 1994 (QJM: monthly journal of the Association of Physicians) with active disease defined as BVAS > 0. Relapse was defined as recurrence of active disease requiring escalation of immunosuppressive treatment from maintenance therapy. Disease duration was calculated as the time from initial diagnosis to study enrolment, while time in remission was defined as the period between latest relapse and study inclusion. Relapse rate was calculated as number of relapses per month since first AAV manifestation.

### Sample acquisition

Peripheral venous blood was drawn once from patients and healthy controls using 2,2′,2″,2″′-(Ethane-1,2-diyldinitrilo)-tetraacetic acid (EDTA) containing tubes as anticoagulant. Samples were processed immediately at room temperature and protected from daylight after acquisition. Measurement of C-reactive protein (CRP) and MDRD-eGFR in the patient group were part of clinical routine. All patients and healthy controls gave written informed consent. The local ethics committee (Medizinische Ethikkommission II der Universität Heidelberg am Universitätsklinikum Mannheim) approved the entire study (reference number 2011–339 N). All experiments were performed in accordance with relevant guidelines and regulations.

### FACS analysis

Fourfold whole blood staining with directly conjugated antibodies for CD4 (Pacific Blue, clone RPA-T4), CD45RA (FITC, clone HI100), CD25 (APC, clone M-A251), CD39 (RPE, clone TU66), CD73 (RPE, clone AD2), and CD26 (RPE, clone M-A261) (all purchased from BD Biosciences, Heidelberg, Germany), was performed on 100 μl of each sample (due to technical reasons, 2 patients could not be analysed by FACS; AAV: n = 29, HC: n = 12). Optimal antibody concentrations were determined by serial dilution. After antibody incubation, red blood cells were lysed using BD FACS lysing solution (BD Biosciences, Heidelberg, Germany) followed by thorough washing with phosphate buffered saline (PBS, Sigma Aldrich) containing 2 mM EDTA (Sigma Aldrich) and 3% (w/v) bovine serum albumine (Serva Electrophoresis GmbH, Heidelberg, Germany) and finally fixation with BD cellFIX solution (BD Biosciences, Heidelberg, Germany). For compensation BD compBeads (BD Biosciences, Heidelberg, Germany) were stained in a similar manner as described above. Flow cytometry was performed on a BD FACS Canto II cytometer using negative and fluorescence-minus-one controls and data analysis was performed using FlowJo software version 5.2 (FlowJo LLC, Ashland, USA).

### RNA isolation from PBMC

PBMC were isolated from whole blood using PolymorphPrep^TM^ (Progen Biotechnik GmbH, Heidelberg, Germany) density centrifugation at 450 g for 40 min as previously described^62^. PBMC were collected on the interface, transferred to a new tube and thoroughly washed. Cell numbers were assessed on a Casy1 TT instrument (Schärfe System, Reutlingen, Germany). RNA was isolated using TRIzol (Invitrogen, Carlsbad, USA) according to the manufacturer’s instructions. After precipitation, the RNA pellet was dissolved in nuclease free water and quality and concentrations were assessed on an Infinite 200 plate reader (Tecan Trading AG, Männedorf, Switzerland). Only RNA isolates with an absorbance ratio (wavelength 260 nm/280 nm) ≥ 2.0 were used in the study (isolate from 1 patient did not meet quality criteria).

### cDNA synthesis, TaqMan Low Density Arrays and qPCR

600 ng of RNA (AAV: n = 20, controls: n = 12) were reversely transcribed for TaqMan low density arrays using the TaqMan® MicroRNA Reverse Transcription Kit and Megaplex^TM^ Pools (Applied Biosystems, Carlsbad, USA). RNA samples (AAV: n = 30, controls: n = 12) were reversely transcribed for qPCR using the High-Capacity cDNA Reverse Transcription Kit with RNase Inhibitor from Applied Biosystems. Reverse transcription was performed for both on a 2720 Thermal Cycler (Applied Biosystems, Carlsbad, USA).

MiR expression was assessed by TaqMan Low Density Arrays (Human Pool A, Applied Biosystems, Carlsbad, USA) using RNU48 and Mamm U6 as endogenous controls. Arrays were run on a 7900HT Fast Real-time PCR System (Applied Biosystems) with ROX as reference dye. Expression analysis was computed by Expression suite software (LifeTechnologies, Carlsbad, USA) applying an auto-threshold method. Cycle threshold (CT) values >32 were excluded from analysis.

Quantitative PCR was performed in triplicate using FAM labelled TaqMan assays and Fast Advanced Master Mix (both purchased from Applied Biosystems, Carlsbad, USA) on a StepOneplus thermocycler (for FIH-1 and HIF-1α mRNA, FIH-1: TaqMan assay Hs 00215495_m1, HIF-1α: TaqMan assay Hs 00153153_m1) or 7900HT Fast Real-time PCR System (for miR-31, hsa-miR-31–5p) setting ROX as reference dye. HPRT1 (TaqMan assay Hs 02800695_m1), and RNU48 (SNORD48, Homo sapiens small nucleolar RNA, C/D box 48) served as endogenous controls for FIH-1, HIF-1α mRNA and miR-31, respectively. Comparative CT values were calculated with Expression suite software (FIH-1 mRNA, LifeTechnologies, Carlsbad, USA), and RQ Manager software (miR-31, Applied Biosystems, Carlsbad, USA) by auto-threshold. Relative quantification was performed by using the ΔΔCT method with healthy controls as normaliser.

### Statistical analysis

Statistics were calculated with Graphpad prism 5 software. Since the data failed to show homoscedasticity and did not meet criteria of Gaussian distribution, non-parametric two-sided Mann-Whitney-U-test was applied for comparison of two groups. Correlation analysis was performed using Spearman’s rank correlation coefficient. All data in figures are given as mean ± standard deviation (s.d.). A p-value ≤ 0.05 was considered as significant. The datasets generated and analysed during the current study are available from the corresponding author on reasonable request.
